# Inequities in working conditions and in access to health care for workers

**DOI:** 10.47626/1679-4435-2025-233

**Published:** 2025-10-06

**Authors:** Sergio Roberto de Lucca, Paula Cristina Moreira Couras da Silva

**Affiliations:** 1 Editor-in-Chief, Revista Brasileira de Medicina do Trabalho; 2 Associate Editor, Revista Brasileira de Medicina do Trabalho

Work, unemployment, and the conditions under which labor activities are performed are recognized as social determinants of health. These factors not only affect quality of life but can also trigger illness processes that compromise the full exercise of the right to work. In this regard, occupational and worker health assumes both a strategic and ethical role: to preserve and promote workers’ health. However, a significant portion of the workforce remains underserved by the health care system, posing a persistent and complex challenge for occupational physicians and other professionals engaged in worker health.

Issue 23.3 of Revista Brasileira de Medicina do Trabalho aims to highlight this multifaceted reality. It features studies such as an investigation involving older adults engaged in subsistence work in Medellín, Colombia, which explored food insecurity and emotional symptoms; research on 100 app-based delivery workers in Campinas, Brazil, who work under precarious conditions and have developed collective strategies to resist the control of digital platforms; and a study of 107 records analyzing the prevalence and complications of COVID-19 among rural workers in Rio Grande do Sul.

Regarding health professionals, a study involving 136 caregivers of patients who had suffered a stroke found significant caregiver burden and impaired quality of life. A review article on burnout among nursing staff reported a dramatic increase (96.4%) in cases between 2014 and 2024, predominantly affecting women in the Southeast region. Another review article examined the frequency of work-related musculoskeletal disorders among physical therapists, with high prevalence of self-reported pain in the lower back, neck, and shoulders. An ecological study based on data from the Brazilian Notifiable Diseases Information System covering the period from 2020 to 2024 reported 289 cases of biological material accidents among primary health professionals, with dental procedures identified as the main context for such occupational accidents.

As for university faculty, a Panamanian study revealed signs of psychological distress, excessive alcohol consumption, and reduced sleep duration among this group. Another study, involving pregnant teachers in the state public school system in Minas Gerais, highlighted emotional challenges and associated factors, such as elevated levels of anxiety and depression among participants.

Focusing on sectors that are less commonly explored in literature, 2 studies examined mining and aviation. The research on psychosocial factors associated with workplace fatigue in the mining sector showed that heavy vehicle operators faced high job demands and low social support - both of which were strongly linked to fatigue. A review on obstructive sleep apnea syndrome in aviation emphasized the importance of medical assessments in aviation, given the high responsibility aviation professionals bear for human lives.

In the field of public policy and occupational health surveillance, 2 review articles addressed occupational cancer risks. The first examined the impact of prolonged exposure to carcinogens in sectors such as construction, mining, and the chemical industry, concluding that replacing harmful substances and implementing effective preventive measures could significantly reduce occupational lung cancer cases. The second review stressed the need for strong public policy regarding skin cancer, considering the large number of outdoor workers exposed to sunlight and ultraviolet radiation. Although progress has been made in workplace sun safety policies, the study highlighted gaps in their effectiveness and called for greater investment in training, communication, and international benchmarking to improve outcomes.

The characteristics of tinnitus among workers exposed to occupational noise were the focus of a systematic review. The studies indicated that tinnitus was most often perceived in both ears, described as high-pitched, with mild to moderate loudness. In light of the growing number of remote workers and the often-overlooked issue of self-care, a study evaluating a multicomponent active break program for office workers emerged as a promising strategy to mitigate these adverse effects.

An article on hazardous duty pay in Brazil’s federal universities for public servants sparked a broader debate about workplace hazards. It argued that this issue should not be reduced to financial compensation for risk, and emphasized that preventive measures and health promotion must take precedence.

Considering hazardous work environments and the toxic organizational cultures that often persist, a review focused on strategies to promote health and well-being in the workplace. Drawing on international models, the authors highlighted 2 benchmark frameworks: Workplace Health Promotion Embedded in Medical Surveillance and Total Worker Health^®^. These models call on organizations to adopt integrated actions combining medical surveillance with psychosocial support to reduce and eliminate risk factors and strengthen stakeholder engagement.

The articles in this issue illustrate the heterogeneity of the labor world and the unequal distribution of the workforce and its vulnerabilities. In this context, occupational physicians and other professionals in worker health face major challenges, including the need for ongoing education to expand their scope of practice and the fight for public policies that reduce inequalities in access and care. This is particularly evident in the distribution of health professionals, as exemplified by an article on the regional disparity in occupational medicine residency positions in Brazil.

Enjoy your reading!

